# Association of Maternal Age and Blood Markers for Metabolic Disease in Newborns

**DOI:** 10.3390/metabo14010005

**Published:** 2023-12-20

**Authors:** Yuhan Xie, Gang Peng, Hongyu Zhao, Curt Scharfe

**Affiliations:** 1 Department of Biostatistics, Yale School of Public Health, New Haven, CT 06510, USA; yuhan.xie@yale.edu (Y.X.); hongyu.zhao@yale.edu (H.Z.); 2 Department of Genetics, Yale School of Medicine, New Haven, CT 06510, USA; 3 Department of Medical and Molecular Genetics, Indiana University School of Medicine, Indianapolis, IN 46202, USA; gangpeng@iu.edu

**Keywords:** maternal age, newborn metabolites, inborn errors of metabolism, public health, newborn screening, precision medicine

## Abstract

Pregnancy at an advanced maternal age is considered a risk factor for adverse maternal, fetal, and neonatal outcomes. Here we investigated whether maternal age could be associated with differences in the blood levels of newborn screening (NBS) markers for inborn metabolic disorders on the Recommended Universal Screening Panel (RUSP). Population-level NBS data from screen-negative singleton infants were examined, which included blood metabolic markers and covariates such as age at blood collection, birth weight, gestational age, infant sex, parent-reported ethnicity, and maternal age at delivery. Marker levels were compared between maternal age groups (age range: 1544 years) using effect size analyses, which controlled for differences in group sizes and potential confounding from other covariates. We found that 13% of the markers had maternal age-related differences, including newborn metabolites with either increased (Tetradecanoylcarnitine [C14], Palmitoylcarnitine [C16], Stearoylcarnitine [C18], Oleoylcarnitine [C18:1], Malonylcarnitine [C3DC]) or decreased (3-Hydroxyisovalerylcarnitine [C5OH]) levels at an advanced maternal age (≥35 years, absolute Cohen’s d > 0.2). The increased C3DC levels in this group correlated with a higher false-positive rate in newborn screening for malonic acidemia (*p*-value < 0.001), while no significant difference in screening performance was seen for the other markers. Maternal age is associated with inborn metabolic differences and should be considered together with other clinical variables in genetic disease screening.

## 1. Introduction

The age of first-time mothers has been increasing in the United States, with the mean maternal age for the first childbirth increasing from 21.4 to 27.1 years from 1970 to 2020 [1,2]. In 2020, nearly 11% of women had their first child at the age of 35 and older compared to 0.25% in 1970 [2,3]. Similar trends have been found worldwide with demographic models predicting further increases in maternal age [4,5,6,7]. Observational research suggests that pregnancy later in life is a risk factor for adverse maternal, fetal, and neonatal outcomes [7,8]. For example, advanced maternal age has been associated with complications such as placenta previa, gestational diabetes mellitus, hypertensive disorders of pregnancy, and higher risk for intra-uterine growth restriction, prematurity, and chromosomal abnormalities [9,10,11]. Maternal metabolism, lifestyle and dietary habits during pregnancy, maternal medical conditions, complications during pregnancy, vaginal delivery versus cesarean section, and environmental stressors such as fetal tobacco exposure have also been found to influence neonatal metabolism and adaptation [12,13,14,15,16,17,18].

In this study, we investigated whether maternal age (MA) could be associated with differences in the blood levels of newborn screening (NBS) markers for metabolic disorders on the Recommended Universal Screening Panel (RUSP) [19]. Accurate detection of these disorders through newborn screening allows for rapid clinical diagnosis and management [20,21]. Importantly, as other covariates including gestational age, birth weight, age at blood collection, infant sex, and parent-reported ethnicity status may concurrently influence neonatal metabolism and development, we accounted for confounding by these covariates and stratified analyses across different maternal age groups. The identified maternal age-related differences in metabolite levels were correlated to false-positive cases in metabolic disease screening. Based on these findings, maternal age is suggested as an important covariate associated with metabolic differences in newborns that should be considered in the interpretation of newborn metabolic screening data and to support the development of algorithms for genetic disease screening.

## 2. Materials and Methods

### 2.1. Data Summary and Preprocessing

We analyzed NBS data for 503,935 screen-negative singleton infants born between 2013 and 2017 and reported by the California NBS program. The data included 41 metabolites measured by MS/MS from newborn dried blood spots [22], 5 additional NBS markers including Galactose-1-phosphate uridyl tansferase (TRA), Thyroid-stimulation hormone (TSH), 17-hydroxyprogesterone (OHP), Immunoreactive trypsinogen (IRT), and T cell receptor excision circles (TREC), and 8 covariates of gestational age (GA), birth weight (BW), age at blood collection (AaBC), infant sex, parent-reported ethnicity, total parenteral nutrition (TPN), transfusion status, and maternal age (MA). Infants reported under the following criteria were removed from analysis: (1) BW less than 1000 g or larger than 5000 g; (2) GA smaller than 28 or larger than 42 weeks; (3) AaBC unknown or before 12 h or after 168 h; (4) total parenteral nutrition (TPN) as unknown or positive; (5) red blood cell transfusion status as unknown or positive; and (6) MA under 15 years or older than 44 years, which resulted in 476,718 infants for analysis (Table S1). Infants with a GA less than or equal to 36 weeks were classified as preterm birth, and those with a GA greater than 36 weeks were classified as term birth. For the ethnicity-stratified analysis, infants with multiple parent-reported ethnicities (17.9%, n = 85,148) and those with unknown ethnicity (2.0%, n = 9542) were removed, resulting in 382,028 (80.1%) newborns classified to only one of 17 racial/ethnic groups (Asian East Indian, Black, Cambodian, Chinese, Filipino, Guamanian, Hawaiian, Hispanic, Japanese, Korean, Laos, Middle Eastern, Native American, Other Southeast Asian, Samoan, Vietnamese, White) (Table S2). In addition, we analyzed data from first-tier NBS false-positive cases for 3 inborn metabolic disorders reported by the California NBS program, which included malonic acidemia (MAL, n = 439), carnitine palmitoyltransferase type II deficiency (CPT-II, n = 51), and 3-Methylcrotonyl-CoA carboxylase deficiency (3MCC, n = 239). The diseases were selected based on 6 corresponding markers identified in the maternal age analysis, of which 3 markers had data available for false-positive screens in the California NBS program. This study was overseen by the institutional review boards at Yale University (protocol #1505015917) and the State of California Committee for the Protection of Human Subjects (protocol #13-05-1236).

### 2.2. Analysis of Maternal Age

To account for the influence of different covariates on newborn metabolite levels, we first studied the correlation between MA and infant sex, gestational age, and parent-reported ethnicity. We then analyzed newborn metabolic profiles across six MA groups with five years per age group. Blood levels of 46 markers in the first MA group (15–19 years) were used as the baseline to explore the gradual changes in marker levels with increasing MA. Effect size analysis using Cohen’s d [23] was performed for each of the 46 markers to calculate marker level differences for the remaining five MA groups in comparison with the baseline group. We used absolute Cohen’s d values larger than 0.2 as the threshold [23,24] for significant differences between the comparison and the baseline groups. We also compared MA-related metabolic differences between the MA group of 35 years or older and the baseline group (15–19 years) [7,16,25].

### 2.3. Analysis of Maternal Age in Relation to Other Variables

Two representative markers with increasing (Palmitoylcarnitine [C16], Malonylcarnitine [C3DC]) and one with decreasing (3-Hydroxyisovalerylcarnitine [C5OH]) levels for MA between 20 and 44 years were selected to investigate the influence on metabolite levels from other covariates. A full list of metabolite names and abbreviations is available at: https://lhncbc.nlm.nih.gov/newbornscreeningcodes/nb/sc/download/analytes.csv (6 June 2023). The three NBS markers were among the metabolites found with the largest changes related to MA identified in the analysis in Section 2.2. Specifically, we compared the changes in metabolite levels related to MA between (1) female and male infants; (2) term and preterm infants; and (3) infants belonging to the four major ethnicity groups including Asian, Black, Hispanic, and White.

### 2.4. Analysis of Maternal Age-Related Differences and False-Positive Results

The three metabolic disorders studied (MAL, CPT-II, and 3MCC) were detected in NBS by elevated marker levels (C3DC, C16, and C5OH). Here we studied whether MA could impact NBS performance in detecting these diseases. In addition to the filtering criteria (1)–(6) described above, this analysis only included infants (n = 405,968) born with a normal birth weight (2500–4000 g) and within the range of a term pregnancy (from 37 to 42 weeks) in order to mitigate the confounding from preterm births. The filtering criteria were consistently applied to the false-positive data, except for the status of red blood cell transfusion, which was not available. We conducted an effect size analysis for all 46 metabolites to compare the MA ≥ 35-year group (n = 90,191 infants) to the baseline group (15–19 years, n = 17,063 infants). For each of the three diseases, we compared the proportion of false positive and screen-negative infants in the MA ≥ 35-year group using the Chi-squared test.

### 2.5. Statistical Analyses and Software

All statistical analyses and visualizations were conducted in R software 4.1.2 with the following packages: dplyr [26], effsize [27], ComplexHeatmap [28], ggplot2 [29], and ggpubr [30]. Distribution of MA related to different clinical variables was visualized in boxplots. The pattern of signature metabolites was visualized using smoothed lines estimated from a generalized additive model [31]. Two sample *t*-tests were performed to check the difference in the mean MA across groups. Comparisons of means in more than two groups were conducted using ANOVA [32]. Effect size analyses were conducted using Cohen’s d values [23]. Patterns of all metabolites with an increase in MA were visualized using heatmaps. Hierarchical clustering was used to classify MA-related profiles across metabolites. A Kolmogorov–Smirnov test [33] was performed to check the enrichment of acylcarnitine (AC) metabolites in the hierarchical clustering results. Proportion tests [34] were performed to check if the proportion of clinical variables in the MA groups was the same.

## 3. Results

### 3.1. Identification of Metabolic Differences Related to Maternal Age

For MA and infant sex, no significant difference was found in the mean MA between male and female infants (*p*-value = 0.98) (Figures S1 and S2). For MA and parent-reported ethnicity, we found a significant difference between MA across the major ethnic groups (Asian, Black, Hispanic, and White) as well as the 17 detailed parent-reported ethnicity groups (*p*-values < 0.001) (Figures S3 and S4). We observed that the Asian and White groups had higher MA compared with Hispanic and Black groups, with the Korean and Japanese subgroups having the highest mean MA (33.9 years and 35.1 years) among all 17 groups. We also identified a significant difference in MA between term and preterm births (*p*-value < 0.001) (Figures S5 and S6). To further check the relationship between MA and GA, we visualized the proportion of preterm births and found a significant difference in the proportion across the six MA groups (*p*-value < 0.001; Figure S7). In addition, we found a significant difference in the proportion of preterm births across MA groups in the Asian, Black, and Hispanic newborn groupings (Figure S8). 

**Figure 1:**
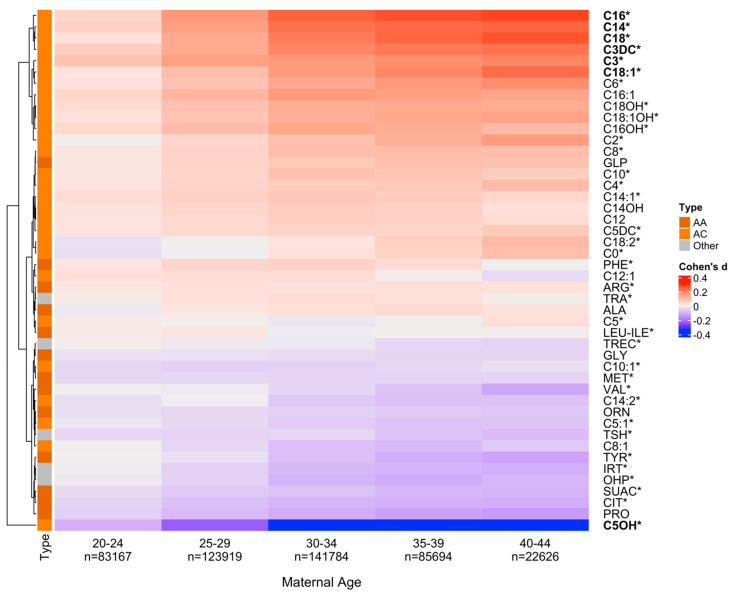
**Newborn metabolite levels and maternal age.** To explore newborn metabolic differences in relation to maternal age (MA), we selected six newborn groups based on MA at delivery with the first group (1519 years, n = 19,528) being defined as a baseline for each metabolite. Effect size differences for the 46 metabolites between each of the five MA groups (2044 years) and the baseline group were calculated. Positive Cohen’s d (in red) indicates increased metabolite levels and negative Cohen’s d (in blue) indicates decreased levels compared to the baseline. Using hierarchical clustering, metabolites were grouped into two clusters of either increasing (at the top) or decreasing (at the bottom) levels compared with the baseline MA group. Seven markers in bold had significant differences between the five MA groups and the baseline group (absolute Cohen’s d > 0.2), including RUSP metabolic disease markers [19] (* label). Acylcarnitines (AC) were enriched in the top cluster of markers with increasing levels. (*p*-value = 0.0088). AA, Amino acid.

The 46 NBS markers clustered into three major groups based on their changing blood levels in relation to MA (Figure 1). The top cluster includes metabolites with increasing levels compared with the baseline group (positive Cohen’s d values; e.g., C16); the bottom cluster includes metabolites with decreasing levels compared with the baseline group (negative Cohen’s d values; e.g., C5OH); while metabolites in between showed relatively smaller absolute Cohen’s d values (e.g., ARG). Several metabolites had non-monotonous patterns such as C0 with initially decreasing and then increasing levels in relation to the increase in MA. Overall, 7 of the 46 markers (Propionylcarnitine [C3], Tetradecanoylcarnitine [C14], C16, Stearoylcarnitine [C18], Oleoylcarnitine [C18:1], C3DC, and C5OH) had significant differences between the five MA groups and the baseline group (absolute Cohen’s d > 0.2). Additionally, a significant enrichment of acylcarnitines was found among the top-ranking metabolites in the hierarchical cluster analysis (*p*-value = 0.0088). 

**Figure 2:**
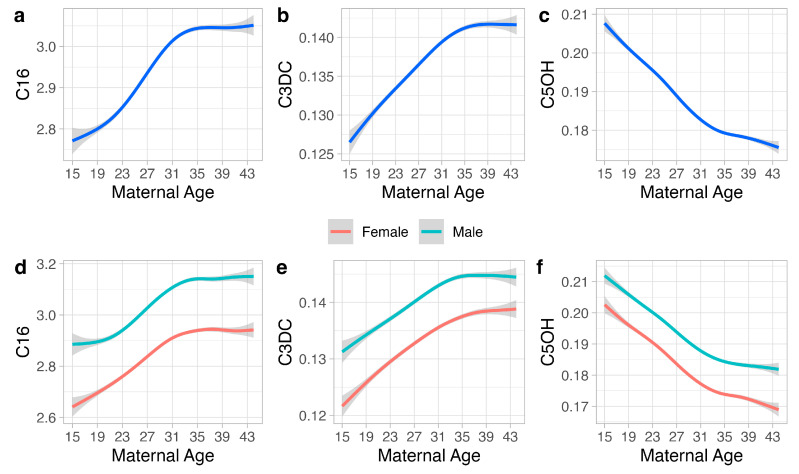

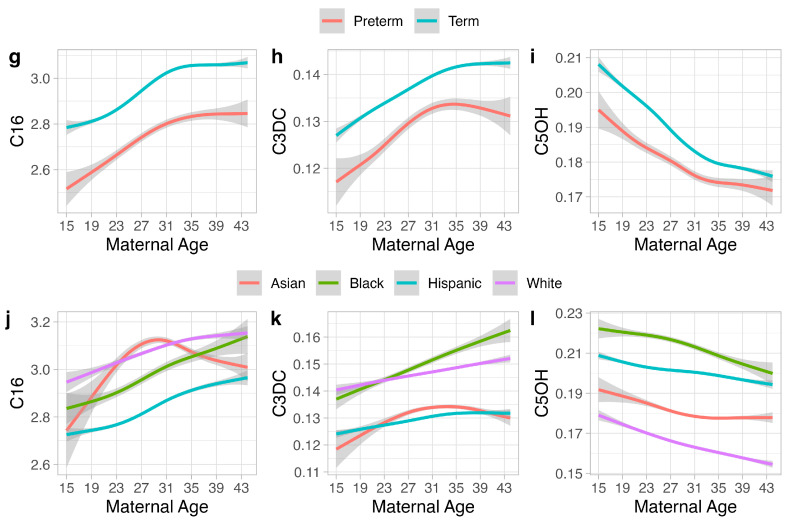
**Maternal age and clinical variables**. The association between maternal age at delivery (1544 years) and three representative metabolites (C16, C3DC, C5OH) (**a**–**c**) and the association between these metabolites, maternal age, and the covariates of infant sex (**d**–**f**), gestational age (**g**–**i**), and parent-reported ethnicity (**j**–**l**) are shown. For each metabolite, the relationship between different maternal ages is shown for male (n = 247,446) and female infants (n = 229,272) (**d**–**f**); preterm (n = 23,541) and term (n = 453,177) (**g**–**i**); Asian (n = 52,642), Black (n = 23,902), Hispanic (n = 184,595), and White infants (n = 120,362) (**j**–**l**). Solid smoothed lines are means estimated from generalized additive models with the shading showing the 95% confidence interval of the mean estimation.

From the seven markers identified with significant differences compared with the baseline group in Figure 1 (absolute Cohen’s d > 0.2), we selected three markers (C16, C3DC, C5OH) to show the metabolic changes associated with MA and other variables, including infant sex, gestational age, and parent-reported ethnicity (Figure 2). The three metabolites are NBS markers for the detection of three metabolic disorders (CPT-II, MAL, and 3MCC) on the RUSP. For C16, mean blood levels initially increased for MA from 15 to 35 years and then plateaued. C16 levels were higher in males compared to females, while term infants exhibited higher C16 levels than preterm infants. White infants had higher C16 levels compared to other groups, while Asian infants showed a distinct trend of initially increasing levels from 15 to 28 years followed by decreasing levels from 28 to 44 years. For C3DC, mean levels showed similar patterns to C16, with the exception of higher C3DC levels in Black and White infants. For C5OH, mean levels monotonously decreased with increasing MA. Infant sex, gestational age, and parent-reported ethnicity all had an influence, with term Black male infants having the highest C5OH mean levels compared to the other groups. Notably, for all three markers, term and male infants had the highest mean levels compared to other groups.

### 3.2. Correlation of Maternal Age-Related Differences to False-Positive Results

**Figure 3:**
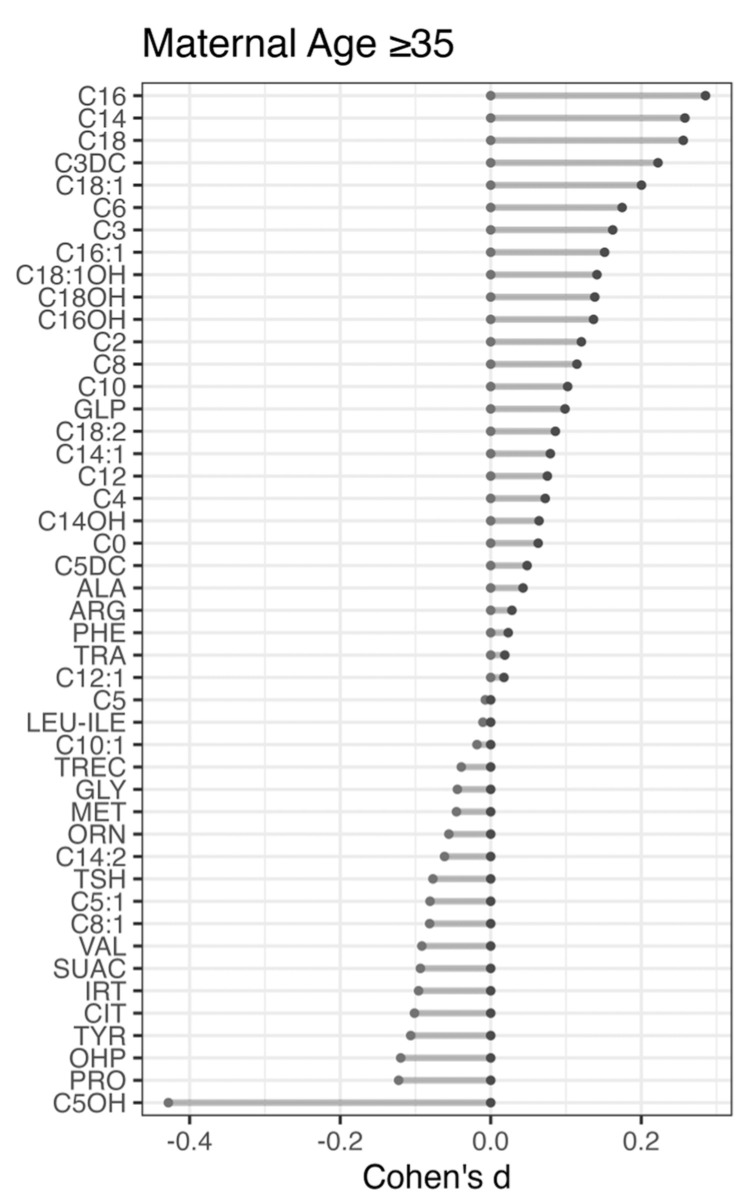
**Newborn metabolic difference in relation to advanced maternal age (≥35 years)**. To identify metabolic differences related to advanced maternal age, 405,968 screen-negative term infants (3742 weeks) with birth weights from 2500 g to 4000 g were grouped into the advanced (≥35 years) and the baseline (1519 years) maternal age groups. Effect size differences (Cohen’s d) for each of the 46 metabolites were calculated between the advanced and the baseline MA group. Positive Cohen’s d values indicate elevated metabolite levels in the advanced MA group. Metabolites are ranked from top to bottom based on Cohen’s d values.

MA-related differences were identified for 13% (6 of 46, Cohen’s d > 0.2) of the metabolites by comparing their levels between the MA group of ≥35 years and the baseline group (Figure 3). These markers (C14, C16, C18, C18:1, C3DC, C5OH) were also identified in our hierarchical clustering analysis (Figure 1) indicating consistency in results. Three of the metabolites identified are RUSP metabolic disease markers (CPT-II, MAL, and 3MCC). Compared to the baseline group, the advanced MA group had elevated levels of the MAL marker C3DC (Cohen’s d = 0.22) and the CPT-II marker C16 (Cohen’s d = 0.29) and decreased level of the 3MCC marker C5OH (Cohen’s d = −0.43). We reasoned that disease markers with an increased level in infants in the MA ≥ 35-year group could also lead to a higher number of false positives, while markers with lower levels could be associated with a lower false-positive rate in this group. To test this hypothesis, we compared the proportion of healthy, screen-negative infants in the advanced MA group (n = 90,191, 22.2%) to the proportion of false-positive cases in that group for each of the three disorders. No significant difference was found in the number of expected versus the number of identified false-positive cases for CPT-II (nine identified, nine expected) and 3MCC (31 identified, 33 expected). For malonic acidemia (MAL), a significantly higher number of false-positive cases was found in the advanced MA group (54 found, 35 expected, *p*-value < 0.001). Considering that early blood collection could influence metabolite levels and NBS false-positive rates [24], MAL false-positives with AaBC < 24 h were removed from this analysis, which confirmed the significantly higher number of false-positive cases in the advanced MA group (52 found, 34 expected, *p*-value < 0.001).

## 4. Discussion

Advanced maternal age is associated with adverse pregnancy outcomes and yet little is known about the influences of maternal age on newborn metabolism. Here we used population-level newborn screening data to study the relationship between maternal age at delivery and newborn metabolite levels and whether maternal age could impact the performance of newborn screening for metabolic disorders [19]. Previous studies have explored the influence of a number of covariates such as gestational age, infant sex, birth weight, age at blood collection, parent-reported ethnicity, season of birth, and nutritional therapy on newborn metabolic profiles [35,36,37,38,39,40,41]. A neonatal metabolome study of dried blood spots retrieved from the Danish Neonatal Screening Biobank found that approximately 16% of the metabolites correlated with gestational age [42]. A study by Australian investigators found that delivery mode, sex, gestational age, and birth weight were associated with specific metabolite levels in cord blood [43]. NBS programs are increasingly using such information in order to reduce false-positive results and increase screening accuracy [44,45,46,47,48,49,50]. Considering the known influence of gestational age, birth weight, age at blood collection, and parent-reported ethnicity on newborn metabolism, we followed a stringent study design and controlled for the influence of these important covariates in the analysis of marker levels between maternal age groups.

A cluster analysis of 46 NBS markers reported for 476,718 screen-negative infants (Table S1) in relation to maternal age showed two large groups of metabolites characterized by either decreasing or increasing levels shortly after birth (Figure 1). We identified significant differences for seven newborn metabolites (absolute Cohen’s d > 0.2) in an effect size analysis of metabolite levels between five maternal age groups (range 20–44 years) in comparison to the baseline group (15–19 years). Six of the seven markers identified were confirmed in a separate analysis comparing newborn metabolite levels between the two groups of advanced maternal age (≥35 years) and teenage maternal age (15–19 years). The six newborn metabolites identified included two short-chain (C3DC, C5OH) and four long-chain (C14, C16, C18, C18:1) acylcarnitines. The enrichment of acylcarnitines in relation to maternal age at delivery (*p*-value = 0.0088) sheds new light on early postnatal metabolic differences. In previous work, ethnicity-related metabolic differences in infants showed larger differences in blood levels of acylcarnitines than of amino acids [51]. In addition to their use in NBS for inborn errors of fatty acid oxidation and energy metabolism, acylcarnitines are increasingly being recognized as biomarkers for a range of diseases such as diabetes, cardiovascular disorders, cancer, and as pharmaceutical agents [52].

To investigate these results, we performed covariate-stratified analyses of maternal age in relation to newborn metabolic profiles. We first considered that metabolic profiles could differ between male and female infants. Infant sex-stratified analyses showed similar cluster patterns for female (Figure S9) and for male infants (Figure S10) and confirmed the same seven acylcarnitine markers identified in the sex-combined analysis (Figure 1). We then studied whether the inclusion of infants with an early AaBC between 12 and 24 h after birth could have an impact on our results. Removing infants with early AaBC from this analysis resulted in the same six (of the seven) metabolites identified for infants in the larger AaBC range (Figure S11). Interestingly, the MA-related metabolites found in this study are different from those identified previously with AaBC-related changes [24], suggesting that MA and AaBC influence different sets of metabolic markers. We also considered that metabolic profiles could differ between parent-reported ethnicity groupings. Ethnicity-stratified analyses revealed distinct metabolic clusters for Asian, Black, Hispanic, and White newborn groups (Figures S12–S15); however, each analysis identified six of the seven markers found in the ethnicity-combined analysis (Figure 1), which supported the robustness of the global analysis.

We then examined the influence on newborn metabolites for several clinical variables (infant sex, gestational age, and ethnicity) in relation to maternal age. Figure 2 shows results from a covariate-stratified analysis of selected metabolites with increasing (C16, C3DC) and with decreasing (C5OH) levels in relation to maternal age (Figure 2). Term infants and male infants had a tendency for higher levels for all three metabolites, while the major ethnicity groups showed distinct metabolite patterns in relation to maternal age. These examples illustrate the variable influences from the different covariates on newborn metabolite levels. Additionally, our analysis identified an overall association between maternal age and prematurity (Figure S5, *p*-value < 0.001), which was found to be significant for the Asian, Black, and Hispanic but not for the White sub-groups (Figure S6). Interestingly, the proportion of preterm births to term births in relation to maternal age varied between different ethnicity groups (Figure S8). The lowest preterm birth rates for Black and Hispanic infants were seen at maternal ages of 20–24 years, while it was shifted to the right for the Asian and White groups (25–29 years). Our findings are consistent with previous studies identifying metabolic differences in relation to parent-reported ethnicity [40,41], suggesting a potential need for assessing ethnicity-related metabolite ranges. These findings also highlight the complex relationship between maternal age, gestational age, infant sex, and parent-reported ethnicity and motivate the development of novel data mining algorithms that incorporate all screening metabolites and covariates in the analysis of newborn screening data.

We hypothesized that the maternal age-related differences identified for 13% of the metabolites (Figure 3) could lead to false-positive results. We selected three diseases detectable using these markers and associated with frequent false-positive screens. Analysis of false-positive cases for one of the diseases revealed maternal age-related differences, which correlated with marker-level differences in the respective MA groups. Infants in the advanced maternal age group (≥35 years) were more likely to be false positive for malonic acidemia (MAL), which correlated with the increased C3DC levels in screen-negatives in this group. In contrast, we did not find higher false-positive rates for CPT-II (marker C16) and 3MCC (marker C5OH) in this group, which was likely due to the smaller number of false positives for these conditions.

Our study had several limitations. First, metabolite levels are influenced by a number of factors such as gestational age (GA), birth weight (BW), and age at blood collection (AaBC) [24,49]. Investigating the relationship between maternal age and newborn metabolites requires a stringent approach that minimizes the influence of other covariates. While residual confounding cannot be fully eliminated, such approaches inherently result in a significant decrease in the sample size and statistical power. For example, after removing infants outside of the defined ranges for GA, BW, and AaBC, only 35% of the MAL false-positive cases (156 of 439) were available in this analysis. Thus, it could be possible that this covariant-stratified analysis has led to an underestimate of the true maternal-age-related effects on newborn metabolism. Second, significant differences in maternal age were found in both the major (N = 4) and the detailed (N = 17) parent-reported ethnicity groupings (Figures S3 and S4). However, the analysis of maternal age and newborn metabolic differences was limited to the four major ethnicity groupings due to the small sample size in some of the detailed sub-groups. The significantly higher maternal age in some Asian-ancestry sub-groups (Korean, Japanese [both *p*-values < 0.001]) could potentially be associated with differences in newborn metabolic patterns. Importantly, GA, BW, and AaBC could vary across different ethnic groups, and there could be combined effects of these variables on metabolite levels. In this work, we performed separate analyses for the influence of each variable in relation to maternal age. Notably, although parent-reported ethnicity data could aid in the interpretation of metabolic screening data, it may not be available to many NBS programs. Third, although infants reported with multiple ethnicity categories were removed from the analysis (~18%, Table S1), this approach is highly limited as the population admixture is often unknown. Additionally, many families do not identify themselves as belonging to predefined ethnicity categories and/or may affiliate with other ancestries [51]. Future studies could explore metabolic differences in cohorts with multiple parent-reported ethnicities to increase statistical power. Fourth, metabolite levels could vary due to multiple factors not evaluated in this study such as seasonal changes in temperature, sample shipping times, or manufacturer kit lot changes [53,54]. Previous studies have shown that smoking during pregnancy could increase the risk of preterm birth and low birth weight [55,56], while breastfeeding and variability in neonatal protein catabolism could influence blood metabolite levels [57]. Newborn metabolism could also be confounded by other risk factors in pregnancies of an advanced maternal age such as placenta previa, hypertensive complications, gestational diabetes mellitus, and other maternal medical history [12,13,15,16,17]. For example, Bass and Taylor found that combining prenatal screening of maternal serum with maternal age could help with detecting fetal disorder (trisomy 18) [58]. However, it may not be feasible to take into account all the factors that could potentially influence metabolite levels. Each NBS laboratory should identify the factors impacting results in their own settings and whether they could contribute to parental anxiety and additional costs for the health care system.

## 5. Conclusions

In conclusion, using population-level newborn screening data, we found that blood markers for newborn metabolic disease were associated with maternal age at delivery. In accordance with previous findings for other covariates [24,41], maternal age did not have a linear correlation with metabolite levels. The association between maternal age and metabolite levels was also dependent on other covariates such as age at blood collection and parent-reported ethnicity. The development of novel data mining models that incorporate newborn metabolic profiles, maternal age, and other clinical variables could further our understanding of metabolite-covariate relationships for improved genetic disease screening and diagnostics.

## Data Availability

The data analyzed in this study are subject to the following licenses/restrictions: The data used in this study were obtained from the California Biobank Program (CBP) under SIS Request 886. The California Department of Public Health is not responsible for the results or conclusions drawn by the authors of this publication. The data can be obtained by others after submitting a new request to the CBP coordinator. Requests to access these datasets should be directed to CaliforniaBiobank@cdph.ca.gov.

## Supplementary Materials

The following supporting information can be downloaded at: https://www.mdpi.com/article/10.3390/metabo14010005/s1, Table S1: Demographics of study population; Table S2. Demographics of infants included in the ethnicity-stratified analyses; Figure S1: Boxplots of maternal age in female infants and male infants; Figure S2: Ethnicity-stratified boxplots of maternal age in female infants and male infants; Figure S3: Boxplots of maternal age in major ethnic groups; Figure S4: Boxplots of maternal age in detailed ethnic groups; Figure S5: Boxplots of maternal age in preterm infants and term infants; Figure S6: Ethnicity-stratified boxplots of maternal age in preterm infants and term infants; Figure S7: Proportion of preterm birth and maternal age; Figure S8: Proportion of preterm birth and maternal age in major ethnic groups; Figure S9: Metabolic analyte levels and maternal age in female infants; Figure S10: Metabolic analyte levels and maternal age in male infants; Figure S11: Newborn metabolite levels and maternal age after removing infants with age at blood collection before 24 hours; Figure S12: Metabolic analyte levels and maternal age in Asian infants; Figure S13: Metabolic analyte levels and maternal age in Black infants; Figure S14: Metabolic analyte levels and maternal age in Hispanic infants; Figure S15: Metabolic analyte levels and maternal age in White infants.
