# Supplementary material for: Association of Maternal Age and Blood Markers for Metabolic Disease in Newborns
Source: Metabolites. 2023 Dec 20;14(1):5. doi: 10.3390/metabo14010005 (PMC10821442; doi:10.3390/metabo14010005)
Supplement: Supplementary file 1 [file metabolites-14-00005-s001.zip › metabolites-2750498-supplementary.pdf]

Supplementary Text S1

Supplementary Tables

	15-19 (N=19528)	20-24 (N=83167)	25-29 (N=123919)	30-34 (N=141784)	35-39 (N=85694)	40-44 (N=22626)	Overall (N=476718)
**Sex**							
F	9347 (47.9%)	40122 (48.2%)	59640 (48.1%)	67935 (47.9%)	41293 (48.2%)	10935 (48.3%)	229272 (48.1%)
M	10181 (52.1%)	43045 (51.8%)	64279 (51.9%)	73849 (52.1%)	44401 (51.8%)	11691 (51.7%)	247446 (51.9%)
**Birth Weight (grams)**							
<1000	0 (0%)	0 (0%)	0 (0%)	0 (0%)	0 (0%)	0 (0%)	0 (0%)
1000-2499	927 (4.7%)	3107 (3.7%)	3952 (3.2%)	4652 (3.3%)	3032 (3.5%)	1003 (4.4%)	16673 (3.5%)
2500-3000	4449 (22.8%)	16460 (19.8%)	21619 (17.4%)	23955 (16.9%)	14876 (17.4%)	4105 (18.1%)	85464 (17.9%)
3001-3500	8576 (43.9%)	36199 (43.5%)	52148 (42.1%)	58433 (41.2%)	34755 (40.6%)	8997 (39.8%)	199108 (41.8%)
3501-4000	4594 (23.5%)	22085 (26.6%)	36217 (29.2%)	41952 (29.6%)	25095 (29.3%)	6376 (28.2%)	136319 (28.6%)
4001-5000	982 (5.0%)	5316 (6.4%)	9983 (8.1%)	12792 (9.0%)	7936 (9.3%)	2145 (9.5%)	39154 (8.2%)
>5000	0 (0%)	0 (0%)	0 (0%)	0 (0%)	0 (0%)	0 (0%)	0 (0%)
**Age at Blood Collection (hours)**							
<12	0 (0%)	0 (0%)	0 (0%)	0 (0%)	0 (0%)	0 (0%)	0 (0%)
12-23	4472 (22.9%)	19281 (23.2%)	28172 (22.7%)	29771 (21.0%)	16378 (19.1%)	3882 (17.2%)	101956 (21.4%)
24-48	14170 (72.6%)	59949 (72.1%)	89699 (72.4%)	104598 (73.8%)	64099 (74.8%)	17060 (75.4%)	349575 (73.3%)
49-72	648 (3.3%)	3054 (3.7%)	4674 (3.8%)	5653 (4.0%)	3948 (4.6%)	1274 (5.6%)	19251 (4.0%)
73-168	238 (1.2%)	883 (1.1%)	1374 (1.1%)	1762 (1.2%)	1269 (1.5%)	410 (1.8%)	5936 (1.2%)
>168	0 (0%)	0 (0%)	0 (0%)	0 (0%)	0 (0%)	0 (0%)	0 (0%)
**Term Status**							
Preterm	1003 (5.1%)	3892 (4.7%)	5619 (4.5%)	6801 (4.8%)	4727 (5.5%)	1499 (6.6%)	23541 (4.9%)
Term	18525 (94.9%)	79275 (95.3%)	118300 (95.5%)	134983 (95.2%)	80967 (94.5%)	21127 (93.4%)	453177 (95.1%)
**Multi Ethnic**							
FALSE	16488 (84.4%)	68989 (83.0%)	102611 (82.8%)	115998 (81.8%)	69302 (80.9%)	18182 (80.4%)	391570 (82.1%)
TRUE	3040 (15.6%)	14178 (17.0%)	21308 (17.2%)	25786 (18.2%)	16392 (19.1%)	4444 (19.6%)	85148 (17.9%)

Table S1. Demographics of study population.

	15-19 (N=16323)	20-24 (N=67851)	25-29 (N=99933)	30-34 (N=112755)	35-39 (N=67434)	40-44 (N=17732)	Overall (N=382028)
**Ethnicity (Major)**							
Asian	211 (1.3%)	2326 (3.4%)	11364 (11.4%)	21291 (18.9%)	13886 (20.6%)	3564 (20.1%)	52642 (13.8%)
Black	1484 (9.1%)	6167 (9.1%)	6953 (7.0%)	5419 (4.8%)	3057 (4.5%)	822 (4.6%)	23902 (6.3%)
Hispanic	12527 (76.7%)	45715 (67.4%)	51447 (51.5%)	43138 (38.3%)	24727 (36.7%)	7041 (39.7%)	184595 (48.3%)
White	2071 (12.7%)	13486 (19.9%)	30009 (30.0%)	42796 (38.0%)	25710 (38.1%)	6290 (35.5%)	120362 (31.5%)
Other	30 (0.2%)	157 (0.2%)	160 (0.2%)	111 (0.1%)	54 (0.1%)	15 (0.1%)	527 (0.1%)
**Ethnicity (Detailed)**							
Asian East Indian	23 (0.1%)	367 (0.5%)	2701 (2.7%)	4792 (4.3%)	1956 (2.9%)	266 (1.5%)	10105 (2.6%)
Cambodian	20 (0.1%)	130 (0.2%)	249 (0.2%)	334 (0.3%)	124 (0.2%)	36 (0.2%)	893 (0.2%)
Chinese	32 (0.2%)	622 (0.9%)	4586 (4.6%)	8526 (7.6%)	6149 (9.1%)	1636 (9.2%)	21551 (5.6%)
Filipino	58 (0.4%)	572 (0.8%)	1630 (1.6%)	2820 (2.5%)	2245 (3.3%)	614 (3.5%)	7939 (2.1%)
Guamanian	1 (0.0%)	11 (0.0%)	12 (0.0%)	4 (0.0%)	10 (0.0%)	2 (0.0%)	40 (0.0%)
Hawaiian	5 (0.0%)	42 (0.1%)	63 (0.1%)	72 (0.1%)	40 (0.1%)	11 (0.1%)	233 (0.1%)
Japanese	0 (0%)	13 (0.0%)	80 (0.1%)	328 (0.3%)	389 (0.6%)	170 (1.0%)	980 (0.3%)
Korean	3 (0.0%)	33 (0.0%)	359 (0.4%)	1519 (1.3%)	1207 (1.8%)	272 (1.5%)	3393 (0.9%)
Laos	11 (0.1%)	70 (0.1%)	103 (0.1%)	133 (0.1%)	57 (0.1%)	27 (0.2%)	401 (0.1%)
Other Southeast Asian	23 (0.1%)	164 (0.2%)	483 (0.5%)	660 (0.6%)	392 (0.6%)	90 (0.5%)	1812 (0.5%)
Samoan	19 (0.1%)	92 (0.1%)	120 (0.1%)	98 (0.1%)	54 (0.1%)	10 (0.1%)	393 (0.1%)
Vietnamese	16 (0.1%)	210 (0.3%)	978 (1.0%)	2005 (1.8%)	1263 (1.9%)	430 (2.4%)	4902 (1.3%)
Black	1484 (9.1%)	6167 (9.1%)	6953 (7.0%)	5419 (4.8%)	3057 (4.5%)	822 (4.6%)	23902 (6.3%)
Hispanic	12527 (76.7%)	45715 (67.4%)	51447 (51.5%)	43138 (38.3%)	24727 (36.7%)	7041 (39.7%)	184595 (48.3%)
Native American	30 (0.2%)	157 (0.2%)	160 (0.2%)	111 (0.1%)	54 (0.1%)	15 (0.1%)	527 (0.1%)
Middle Eastern	44 (0.3%)	595 (0.9%)	1651 (1.7%)	1797 (1.6%)	1042 (1.5%)	235 (1.3%)	5364 (1.4%)
White	2027 (12.4%)	12891 (19.0%)	28358 (28.4%)	40999 (36.4%)	24668 (36.6%)	6055 (34.1%)	114998 (30.1%)

Table S2. Demographics of infants included in the ethnicity-stratified analyses. In ethnicity-related analyses, infants with multiple parent-report ethnicities and with unknown ethnicity were removed. In major ethnic group

analyses, infants were grouped into Asian, Black, Hispanic, White, and Other (Native American). In detailed ethnic group analyses, Asian infants were further classified into 13 groups including Asian East Indian, Cambodian, Chinese, Filipino, Guamanian, Hawaiian, Japanese, Korean, Laos, other Southeast Asian, Samoan, and Vietnamese, and White infants were further classified into Middle Eastern and White. Native American was also included. In total, there are four major ethnic groups and 17 detailed ethnic groups.

Supplementary Figures

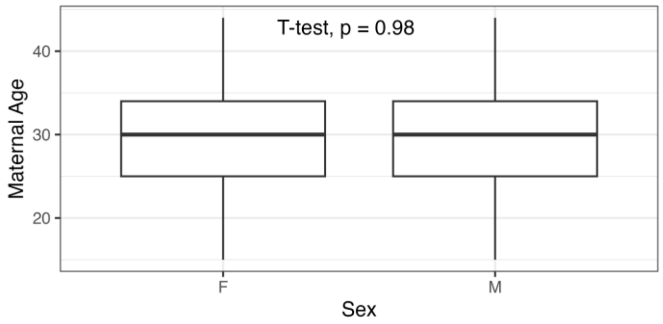

Figure S1. Boxplots of maternal age in female infants and male infants. There is no significant difference between the mean maternal age of female infants and male infants (T-test *p*-value=0.98).

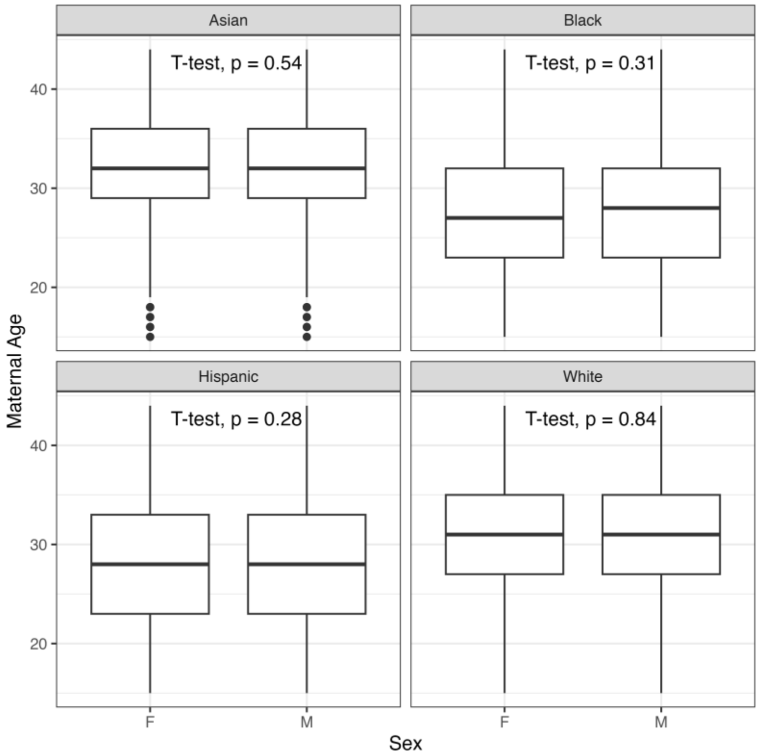

Figure S2. Ethnicity-stratified boxplots of maternal age in female infants and male infants. There is no significant difference in mean maternal age between female infants and male infants in stratified analyses of four major ethnic groups (Asian *p*-value=0.54; Black *p*-value=0.31; Hispanic *p*-value=0.28; White *p*-value=0.84).

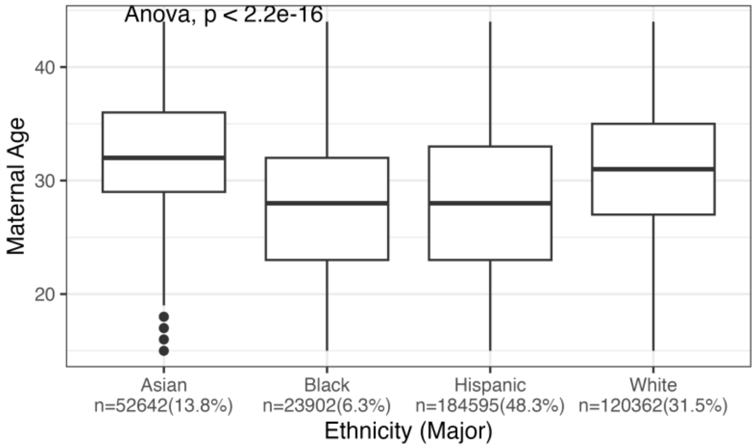

Figure S3. Boxplots of maternal age in major ethnic groups. There is a significant difference between the mean maternal age of all major ethnic groups (ANOVA *p*-value<2.2e-16).

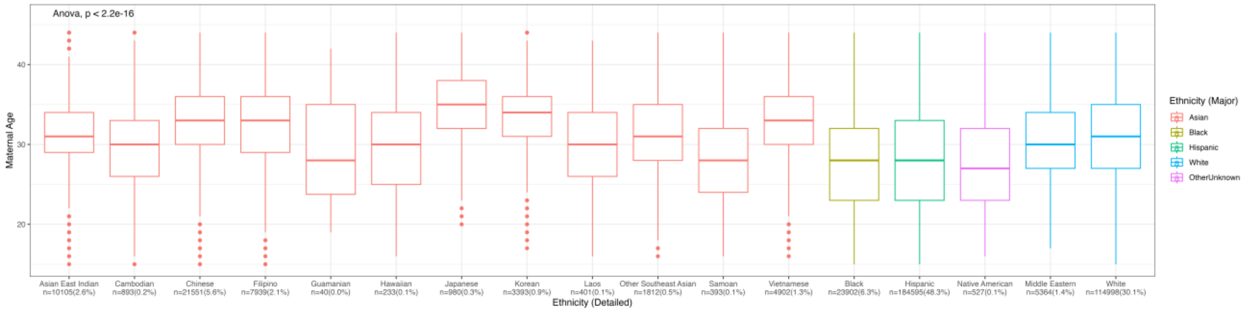

Figure S4. Boxplots of maternal age in detailed ethnic groups. There is a significant difference between the mean maternal age of all detailed ethnic groups (ANOVA *p*-value<2.2e-16).

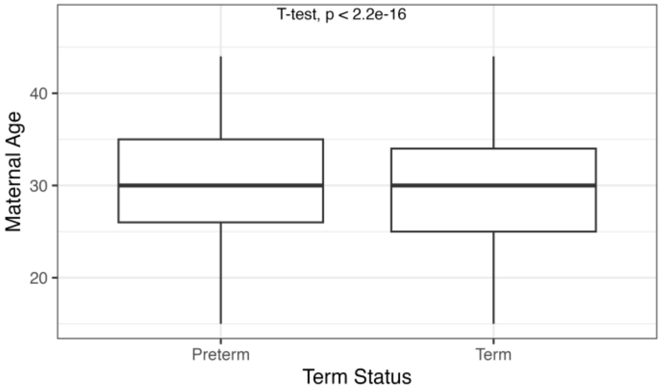

Figure S5. Boxplots of maternal age in preterm infants and term infants. There is a significant difference in mean maternal age between preterm infants and term infants (T-test *p*-value<2.2e-16).

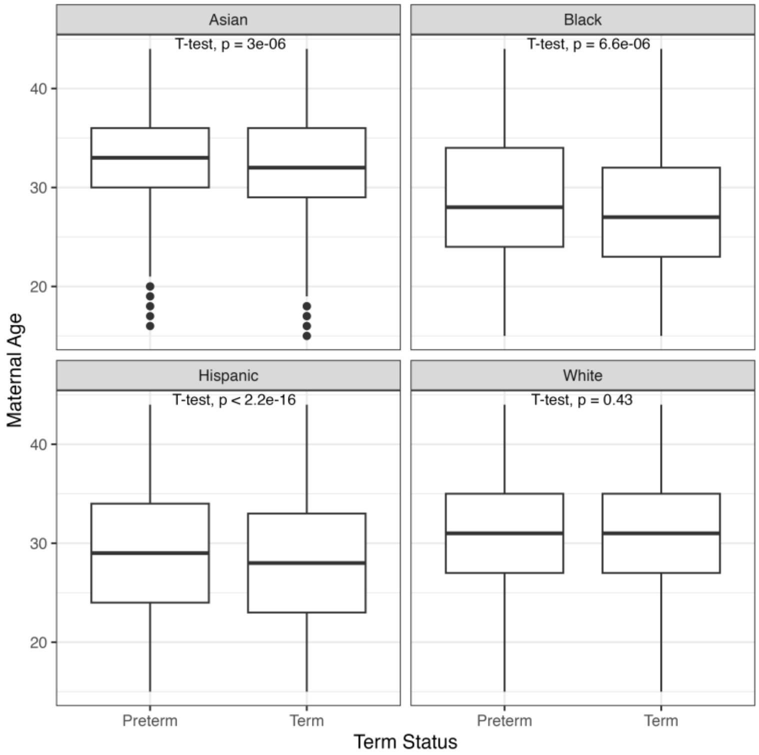

Figure S6. Ethnicity-stratified boxplots of maternal age in preterm infants and term infants. There is a significant difference in mean maternal age between preterm infants and term infants in Asian, Black, and Hispanic infants (Asian *p*-value=3e-6; Black *p*-value=6.6e-6; Hispanic *p*-value<2.2e-16; White *p*-value=0.43).

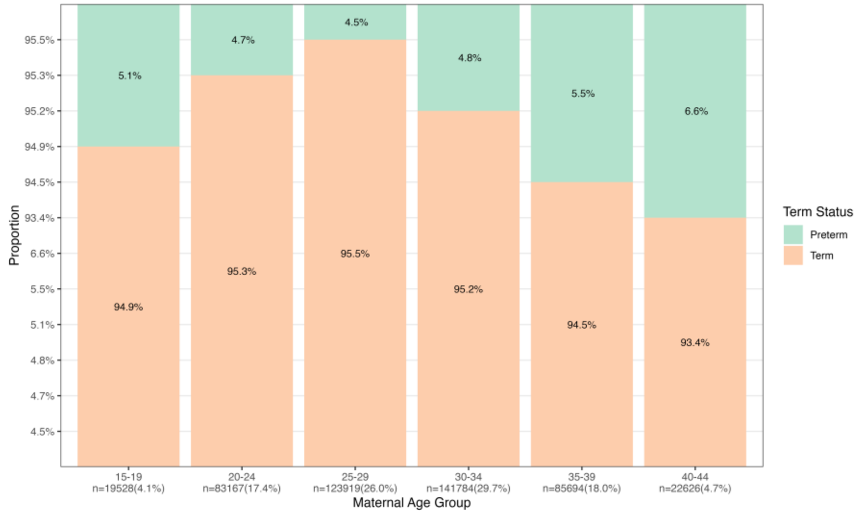

Figure S7. **Proportion of preterm birth and maternal age**. The proportion of preterm birth first decreases and then increases with the increase in maternal age for the overall analysis.

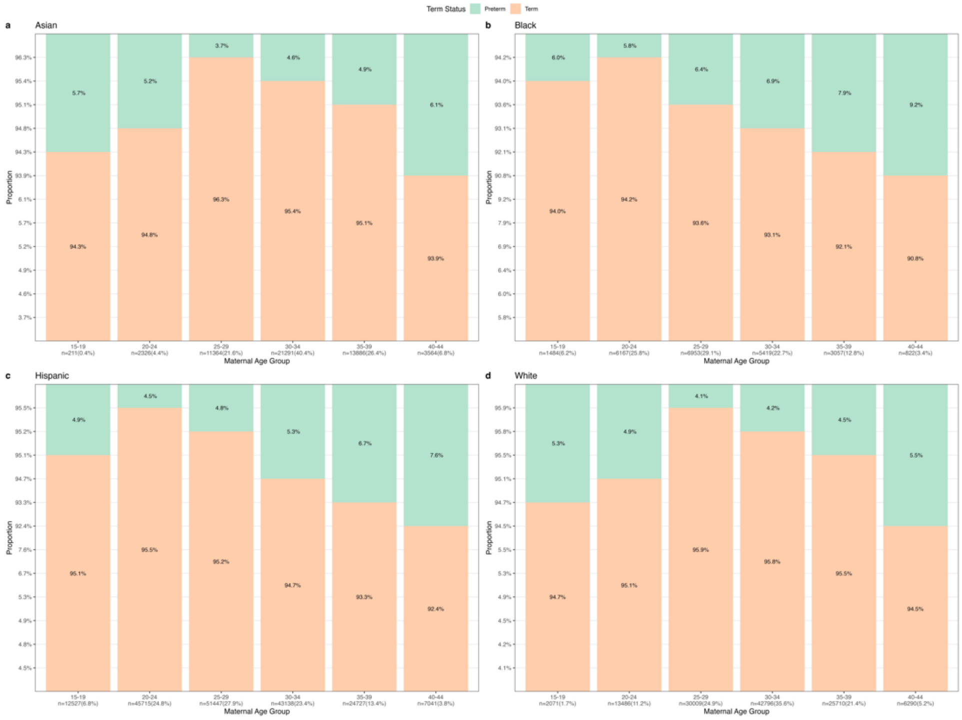

Figure S8. Proportion of preterm birth and maternal age in major ethnic groups. The proportion of preterm births first decreases and then increases with the increase in maternal age. There is a significant difference between the proportion of different maternal age groups in the 4 major ethnic groups. (Proportion test: Asian *p*-value=1.5e-8; Black *p*-value=7.83e-5; Hispanic *p*-value<2.2e-16; White *p*-value=4.7e-8)

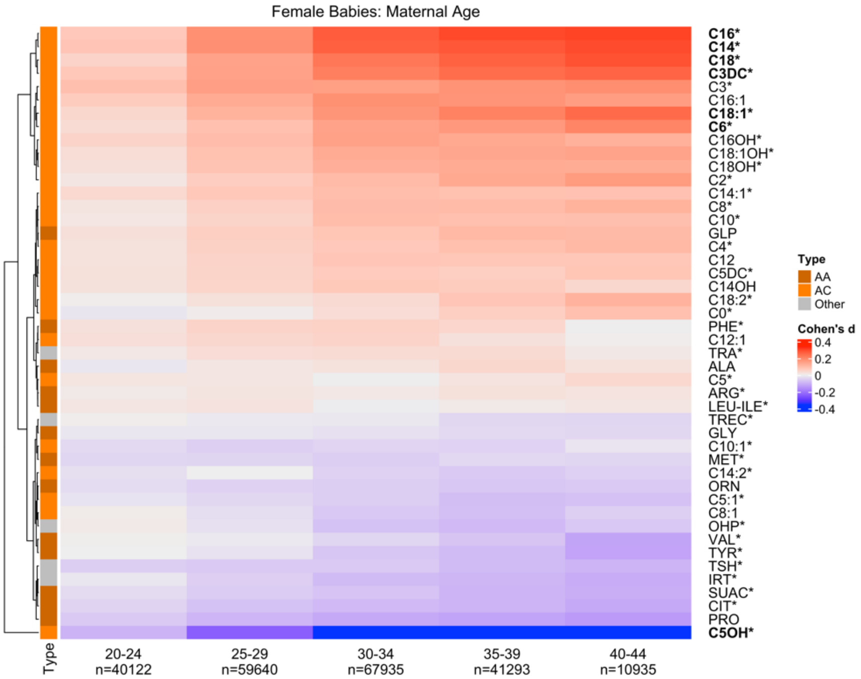

Figure S9. Metabolic analyte levels and maternal age in female infants. Effect size differences (Cohen's d) for all 46 metabolites between each of the five maternal age groups (20–44 years) and the baseline (15–19 years) were calculated. Positive Cohen's d (in red) indicates elevated metabolite levels, and negative Cohen's d (in blue) indicates decreased levels compared to the baseline. Hierarchically clustering was used to group metabolites into two clusters of either increasing (at the top) or decreasing (at the bottom) levels compared with the baseline group. Seven markers in bold showed significant differences between at least one of the seven maternal age groups and the baseline group (absolute Cohen's d>0.2), including NBS markers for the metabolic disorders on the RUSP [19] (labeled with *). Acylcarnities, AC. Amino acid, AA.

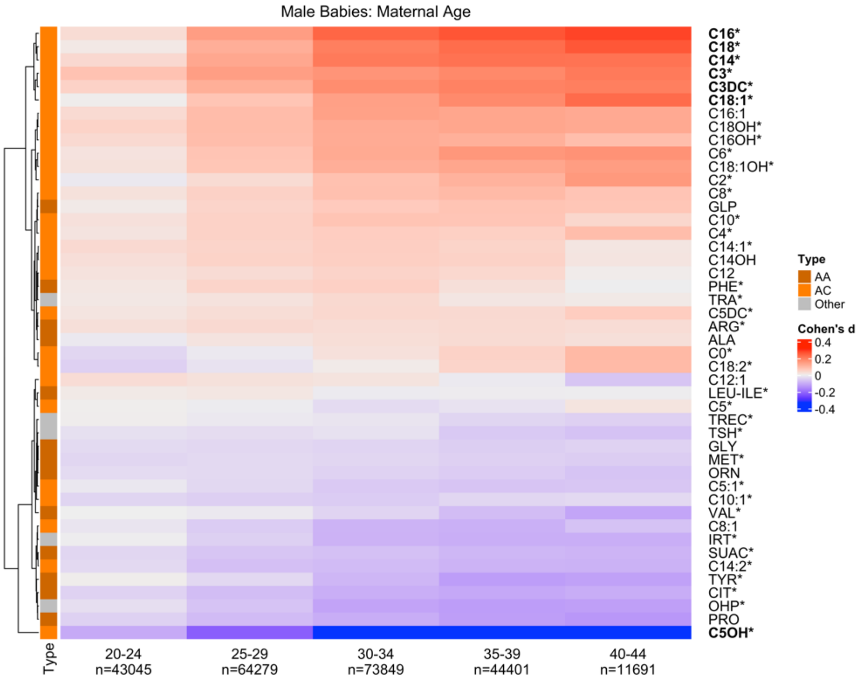

Figure S10. Metabolic analyte levels and maternal age in male infants. Effect size differences (Cohen's d) for all 46 metabolites between each of the five maternal age groups (20–44 years) and the baseline (15–19 years) were calculated. Positive Cohen's d (in red) indicates elevated metabolite levels, and negative Cohen's d (in blue) indicates decreased levels compared to the baseline. Hierarchically clustering was used to group metabolites into two clusters of either increasing (at the top) or decreasing (at the bottom) levels compared with the baseline group. Seven markers in bold showed significant differences between at least one of the seven maternal age groups and the baseline group (absolute Cohen's d>0.2), including NBS markers for the metabolic disorders on the RUSP [19] (labeled with *). Acylcarnities, AC. Amino acid, AA.

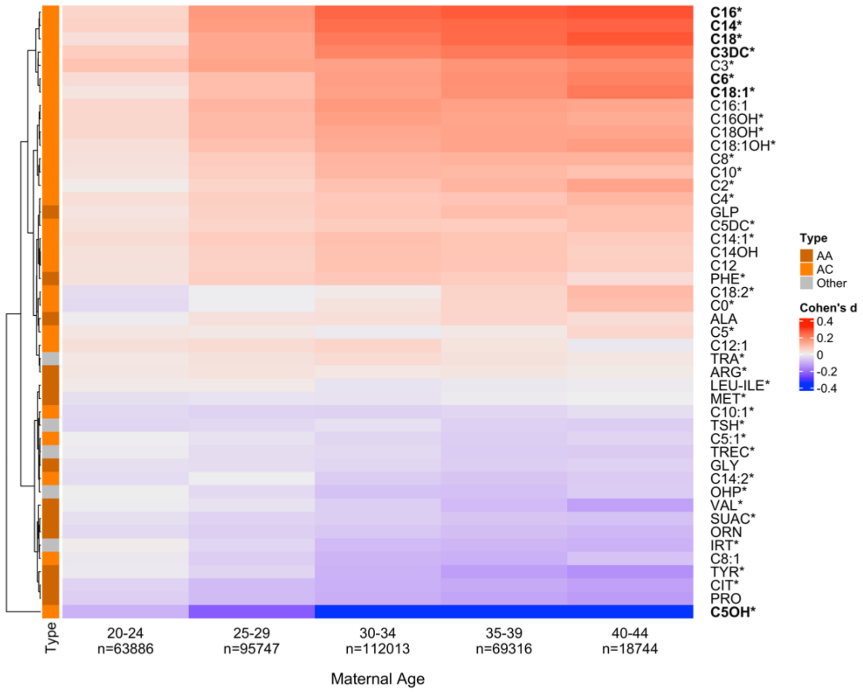

Figure S11. Newborn metabolite levels and maternal age after removing infants with age at blood collection before 24 hours. Effect size differences (Cohen's d) for all 46 metabolites between each of the five MA groups (20–44 years) and the baseline (15–19 years) were calculated. Positive Cohen's d (in red) indicates elevated metabolite levels, and negative Cohen's d (in blue) indicates decreased levels compared to the baseline. Hierarchically clustering was used to group metabolites into two clusters of either increasing (at the top) or decreasing (at the bottom) levels compared with the baseline MA group. Seven markers in bold showed significant differences between at least one of the five MA groups and the baseline group (absolute Cohen's d > 0.2), including NBS markers for metabolic disorders on the RUSP [19] (labeled with *). Acylcarnities (AC) were enriched in the top cluster of markers with increasing levels. (p-value = 0.0088). AA,Amino acid.

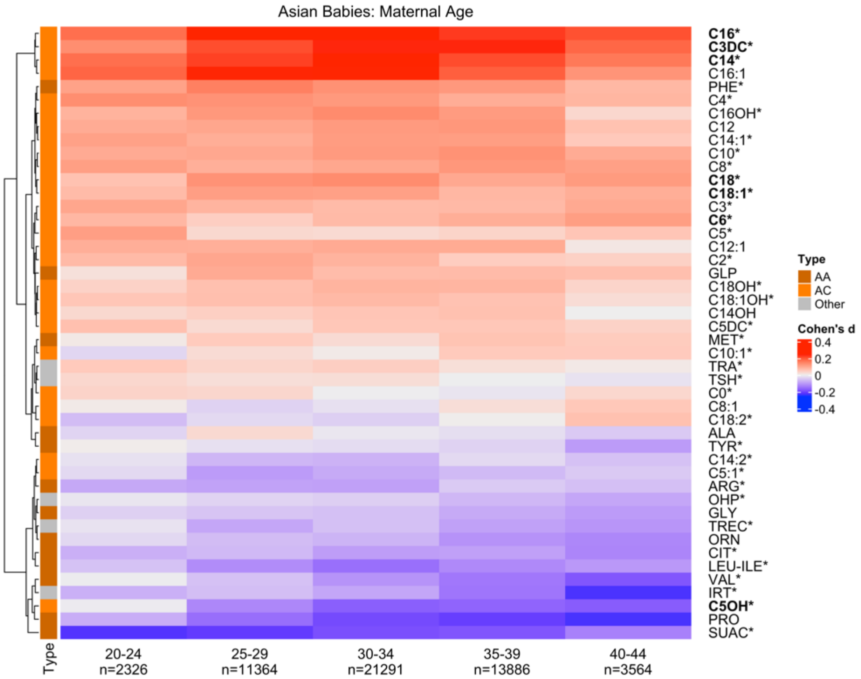

Figure S12. Metabolic analyte levels and maternal age in Asian infants. Effect size differences (Cohen's d) for all 46 metabolites between each of the five maternal age groups (20–44 years) and the baseline (15–19 years) were calculated. Positive Cohen's d (in red) indicates elevated metabolite levels, and negative Cohen's d (in blue) indicates decreased levels compared to the baseline. Hierarchically clustering was used to group metabolites into two clusters of either increasing (at the top) or decreasing (at the bottom) levels compared with the baseline group. Seven markers in bold showed significant differences between at least one of the seven maternal age groups and the baseline group (absolute Cohen's d>0.2), including NBS markers for the metabolic disorders on the RUSP [19] (labeled with *). Acylcarnities, AC. Amino acid, AA.

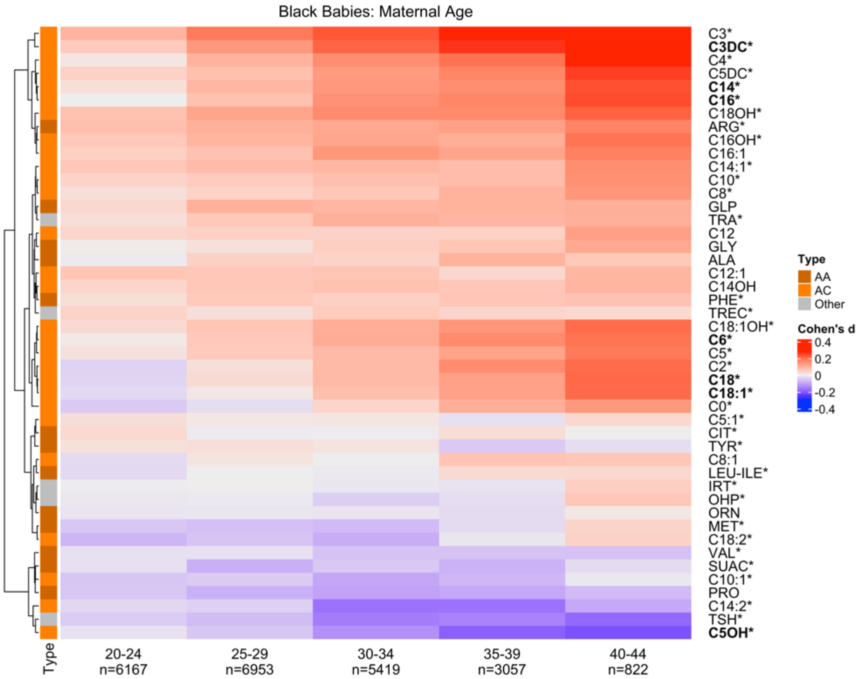

Figure S13. Metabolic analyte levels and maternal age in Black infants. Effect size differences (Cohen's d) for all 46 metabolites between each of the five maternal age groups (20–44 years) and the baseline (15–19 years) were calculated. Positive Cohen's d (in red) indicates elevated metabolite levels, and negative Cohen's d (in blue) indicates decreased levels compared to the baseline. Hierarchically clustering was used to group metabolites into two clusters of either increasing (at the top) or decreasing (at the bottom) levels compared with the baseline group. Seven markers in bold showed significant differences between at least one of the seven maternal age groups and the baseline group (absolute Cohen's d>0.2), including NBS markers for the metabolic disorders on the RUSP [19] (labeled with *). Acylcarnities, AC. Amino acid, AA.

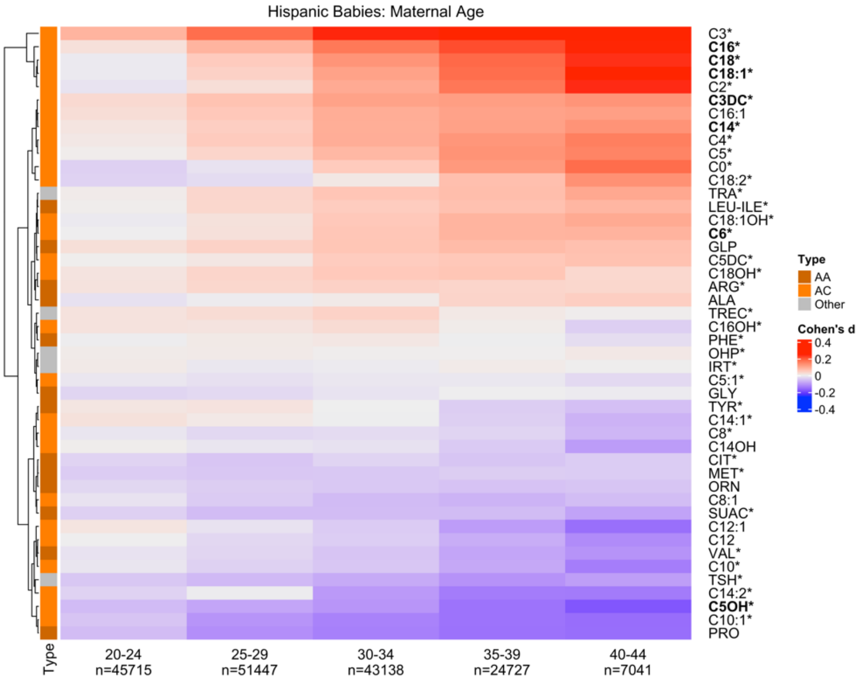

Figure S14. Metabolic analyte levels and maternal age in Hispanic infants. Effect size differences (Cohen's d) for all 46 metabolites between each of the five maternal age groups (20–44 years) and the baseline (15–19 years) were calculated. Positive Cohen's d (in red) indicates elevated metabolite levels, and negative Cohen's d (in blue) indicates decreased levels compared to the baseline. Hierarchically clustering was used to group metabolites into two clusters of either increasing (at the top) or decreasing (at the bottom) levels compared with the baseline group. Seven markers in bold showed significant differences between at least one of the seven maternal age groups and the baseline group (absolute Cohen's d>0.2), including NBS markers for the metabolic disorders on the RUSP [19] (labeled with *). Acylcarnities, AC. Amino acid, AA.

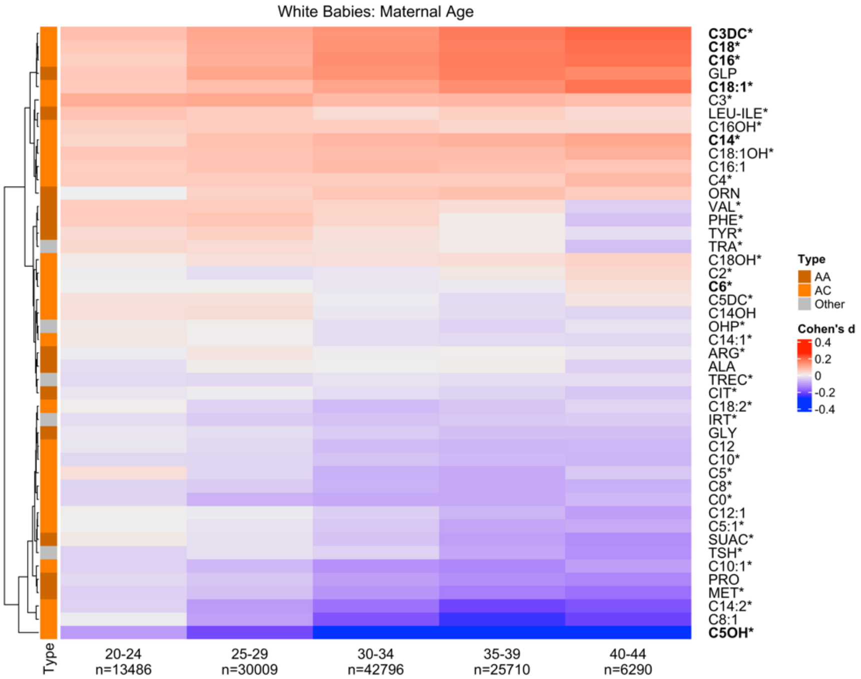

Figure S15. Metabolic analyte levels and maternal age in White infants. Effect size differences (Cohen's d) for all 46 metabolites between each of the five maternal age groups (20–44 years) and the baseline (15–19 years) were calculated. Positive Cohen's d (in red) indicates elevated metabolite levels, and negative Cohen's d (in blue) indicates decreased levels compared to the baseline. Hierarchically clustering was used to group metabolites into two clusters of either increasing (at the top) or decreasing (at the bottom) levels compared with the baseline group. Seven markers in bold showed significant differences between at least one of the seven maternal age groups and the baseline group (absolute Cohen's d>0.2), including NBS markers for the metabolic disorders on the RUSP [19] (labeled with *). Acylcarnities, AC. Amino acid, AA.
